# Perianal ultrasound (PAUS): visualization of sphincter muscles and comparison with digital-rectal examination (DRE) in females

**DOI:** 10.1186/s12905-021-01387-1

**Published:** 2021-06-18

**Authors:** Miriam Hölscher, Charlotte Gräf, Anna-Lena Stickelmann, Elmar Stickeler, Laila Najjari

**Affiliations:** Department of Gynecology and Obstetrics, Medical Faculty, RWTH Aachen University, Pauwelsstraße 30, 52074 Aachen, Germany

**Keywords:** Perianal ultrasound, Digital rectal examination, Anal sphincter muscles, Female fecal incontinence

## Abstract

**Background:**

The aim of this study was to determine the reproducibility and tolerance of perianal ultrasound (PAUS) and detect differences in sphincter muscles between various measuring positions and different maneuvers. PAUS was compared to digital-rectal examination (DRE) to see if sphincter contraction is visible and gradable in ultrasound volumes.

**Methods:**

Fifty women underwent a medical history, DRU and PAUS by two uro-gynecologists in a prospective trial. PAUS volumes were measured via different parameters in different maneuvers. Examiners’ DRE impressions of sphincter tone were scaled with the DRESS-score. All patients completed a questionnaire.

**Results:**

Thirty-five patients with complete PAUS and DRE were included in the study. Fifteen patients were excluded due to poor ultrasound volume quality or sphincter defects. Comparison of sphincter muscle thickness at different positions in PAUS showed significant differences between 6 and 12 o’clock positions (12 > 6 o’clock) and diameters (horizontal > vertical). No difference was found between the examiners. In comparison of rest and contraction only the vertical diameter changed. There was a negative but not significant correlation between PAUS measurements and DRESS-scores. Twenty-six patients completed the questionnaire that revealed women preferred PAUS over DRE.

**Conclusion:**

PAUS is a reproducible and good tool to visualize the anal canal. It is comfortable for patients and easily handled by examiners. Sphincter muscle contraction is iso-volumetric. Vertical diameter changes during contraction leading the anal canal change its shape to oval due to external influence. PAUS is the ideal additional tool to visualize relevant structures that are palpable on DRE.

## Background

Fecal incontinence (FI) increases in aging population, negatively impacts quality of life and leads to social isolation [1, 2]. Women suffer from FI more commonly than men with prevalence rates in women of 2–15% [3–7]. There are several risk factors for FI in women including obstetric trauma with perineal tears or sphincter lesions [1, 8].

Imaging tools to investigate FI and pelvic floor disorders are endoanal ultrasound (EAUS), perineal ultrasound (PUS) and endoanal MRI (eaMRI). EAUS is the goldstandard for sphincter muscle complex imaging and detection of sphincter lesions [9]. Disatvantage of EAUS is the invasive assessment with introducing a probe in the anal canal and therefore perceived as uncomfortable, especially postpartum [10].

PUS is a non-invasive ultrasound examination, which investigates the perineal area and anal canal by placing the ultrasound probe on the perineum. Advantage of this examination is that it is easy to perform, low cost, easily available and has good patient acceptance [11, 12].

PUS is mostly used in the assessment of the urinary tract to evaluate urine incontinence and detect pathologies. Less frequently, PUS is used in the assessment of anal canal complex in patients with FI [8, 11]. Depending on literature EAUS has more reliability in detecting irregularities than PUS, other studies show a strong significant correlation between both modalities [13, 14].

The goldstandard for the assessment of the sphincter tone is the digital-rectal examination (DRE), other modalities are manometry and electromyography [15]. DRE is a widely available simple clinical tool in the diagnosis of anorectal disorders by using the feeling of the examiners finger to identify structural and functional abnormalities. A disadvantage of DRE is that it is a subjective assessment and the impression of sphincter tone can be differerent between examiners. Although there are standardized ratings for sphincter tone assessment like the digital rectal exam scoring system (DRESS) to make it comparable, it is still a subjective impression [16].

Perianal ultrasound (PAUS) is an upcoming non-invasive imaging modality in the assessment of rectal disorders. The probe is located on the anal area. PAUS is also low cost, ultrasound systems are widely available and images can be evaluated objective. However there is scarce information about the reproducibility of images, sphincter thickness and patients’ acceptance.

Anatomically the internal anal sphincter (IAS) is a smooth muscle, external anal sphincter (EAS) and levator ani (LA) muscles are striated. All three anatomical structures are important parts of the complex sphincter system for fecal continence [17]. Physiologically striated muscles can be contracted voluntarily in contrast to smooth muscles [1]. However investigations in the visualization and grading of these muscle contractions by ultrasound are rare.

The aim of this study was to demonstrate that PAUS is a reproducible imaging modality among different examiners and produces diagnostic relevant images of the anal canal. In addition, we investigated differences in sphincter muscles between different measuring positions and between different maneuvers. Furthermore we compared PAUS with DRE to see if sphincter contraction is visible and gradable in ultrasound pictures. Lastly, sought to gauge the patients’ acceptance of PAUS as a diagnostic tool.

## Methods

This study was performed as a prospective trial design according to the Declaration of Helsinki and approved by our local ethics commission (Reference number EK085/11).

Between April and December 2016 we examined 50 women during their visit in the Interdisciplinary Continence Center in our hospital. Reasons for the visit of these patients included urinary incontinence or pelvic organ prolapse, but not fecal incontinence or defecation problems.

All women were asked about their medical history and underwent a clinical examination, including perineal ultrasound (PUS), perianal ultrasound (PAUS) and digital rectal examination (DRE).

PAUS and DRE were performed by two independent specialized doctors, in a center with DEGUM (Deutsche Gesellschaft für Ultraschall in der Medizin e.V.) standard qualification and AGUB certificate (Arbeitsgemeinschaft für Urogynäkologie und plastische Beckenbodenrekonstruktion e.V.). PUS was used to rule out anomalies of the pelvic floor such as fistulas, cysts and organ prolapse. Patients were examined in dorsal recumbent position. Examiners operated independently without knowledge of the others results. The examiners first completed PAUS and then performed DRE.

**Fig. 1:**
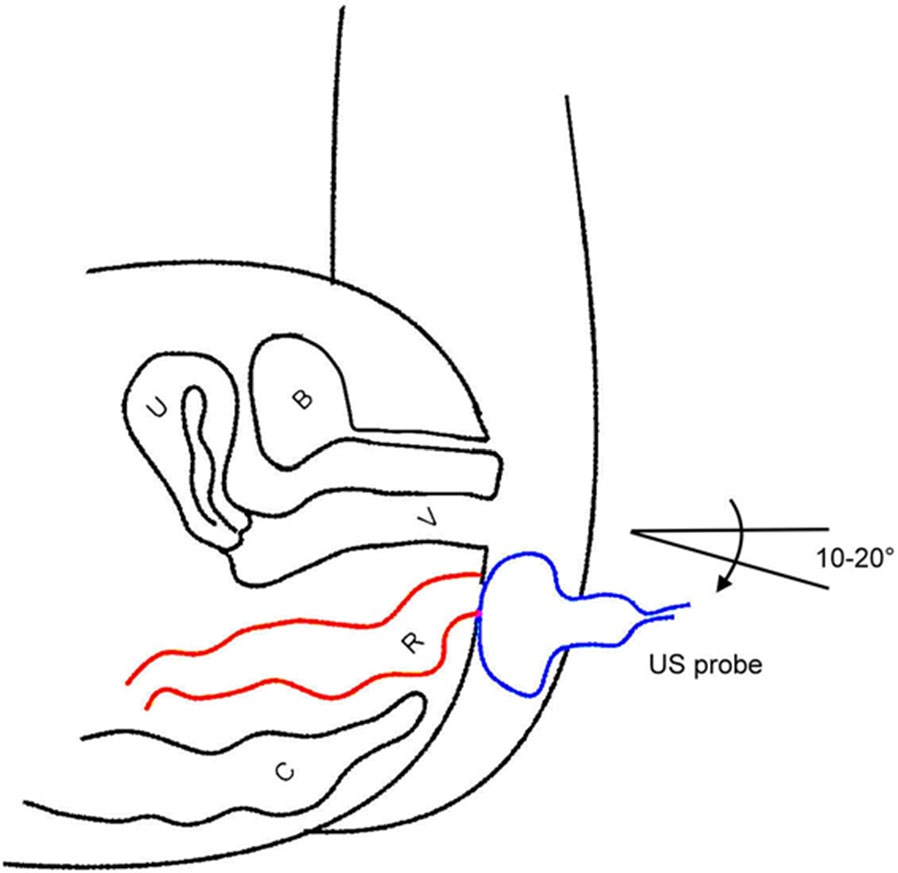
Position of the ultrasound probe for PAUS examination in women in dorsal recumbent position. The probe (3.5–5 MHz) is placed on the anal area and tilt 10°–20° in ventral direction. B = Bladder, U = Uterus, V = Vagina, R = Rectum, C = Coccyx

For PAUS an E8 Voluson ultrasound system (GE Healthcare Ultrasound, Zipf, Austria) was used with a perineal probe (3.5–5 MHz) covered by a condom. The probe was placed with minimal pressure on the anal opening and was tilt 10°–20° in ventral direction (Fig. 1).

**Fig. 2:**
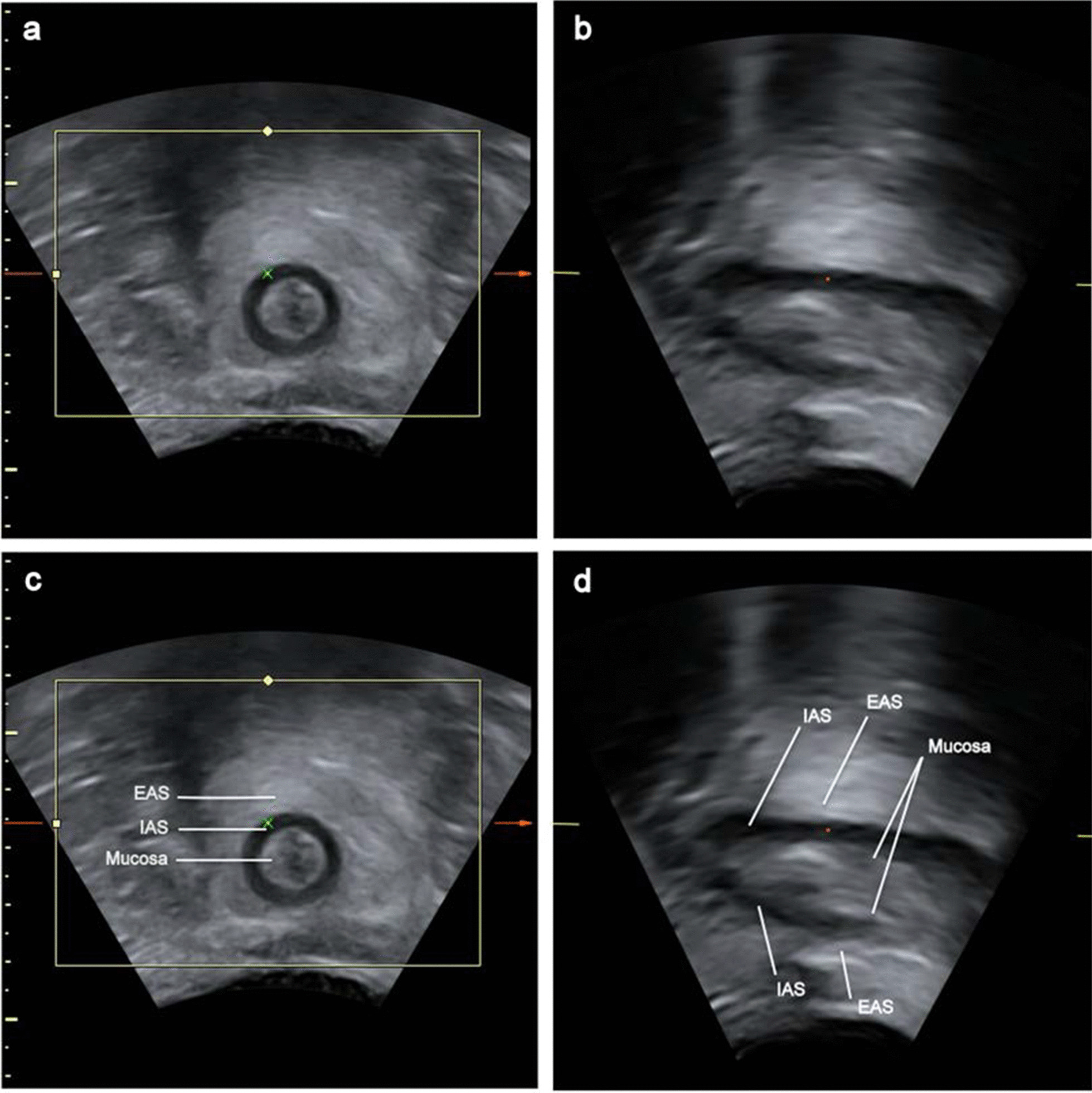
Perianal ultrasound (PAUS) of the anal canal at rest. **a** + **c** Coronal plane, **b** + **d** midsagittal plane

**Fig. 3:**
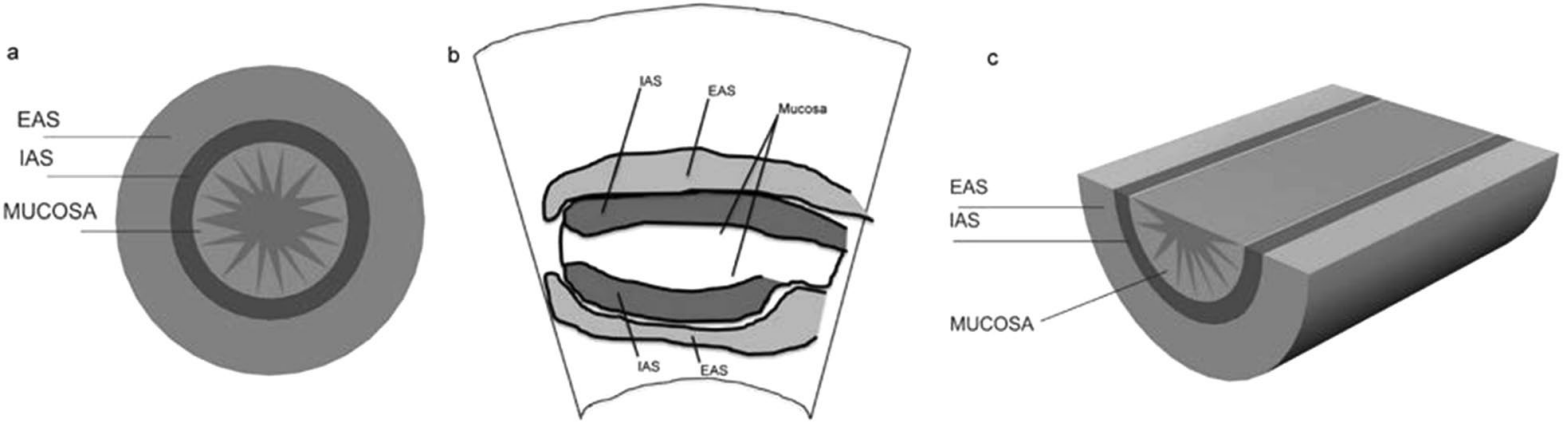
Schematic representation of with PAUS imaged structures. **a** Coronal plane, **b** midsagittal plane, **c** axial plane

PAUS B-mode pictures and 3D/4D-volumes were done standardly during pelvic floor rest and contraction (Figs. 2, 3).

DRE was also performed at rest and during contraction. Examiners assigned scores for resting pressure and contraction pressure with the digital rectal exam scoring system (DRESS) (Score from 0 to 5). A resting score of 0 indicates no discernable tone at rest and an open anal canal. A score of 3 is normal and a score of 5 indicates very high pressures and a tight anal canal. Analog to this the contraction score contains values for the increasing tone during contraction. Zero is associated with no discernable tone increase with contraction effort. Three is normal. A score of 5 indicates a very strong contraction almost painful for the examiner [16].

**Fig. 4:**
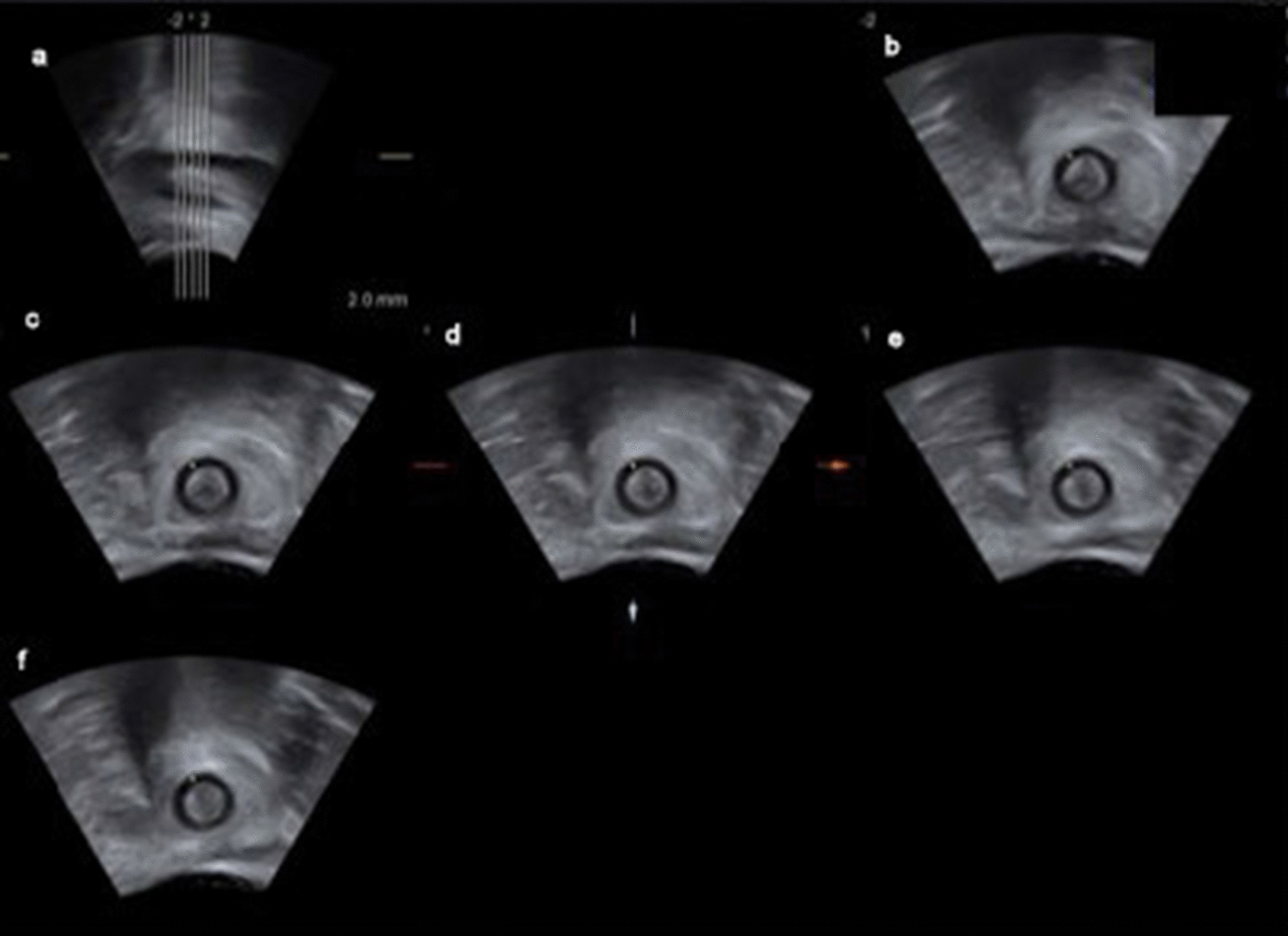
Perianal tomographic ultrasound imaging (TUI) of the anal canal. TUI slices with a width of 2 mm. **a** Midsaggital plane with marked TUI slices, followed by the 5 slices in coronal plane, beginning with the lowermost slice (**b**) where external (EAS) and internal sphincter muscles (IAS) have been both visible completely for the first time starting from the anocutaneous transition zone moving in cranial direction. **c** Slice 2 mm cranial of **b**. **d** 2 mm cranial of **c**, slice where all measurements were done. **e** 2 mm cranial of **d**, **f** 2 mm cranial of **e**, most cranial slice where EAS and IAS are still both seen completely

Further investigations of the ultrasound images were completed by one examiner using the 4D View software (GE Healthcare). Tomographic ultrasound imaging (TUI) was used on the 3D/4D volumes (Fig. 4).

Lowermost slice was defined as the slice where external anal sphincter muscle (EAS) and internal anal sphincter muscle (IAS) have been seen both completely for the first time starting from the anocutaneous transition zone moving in cranial direction. In cranial direction four slices with 2 mm interslice distance were set. Measurements were done in a defined plane two TUI slices (= 4 mm) cranial of the lowermost slice. This was done for standardization of measurements in all patients and to make sure that both sphincter muscles are completely in the plane and rule out or reduce any irregularities, inaccuracies or uncertainties with this safety distance of 4 mm from the edge. Also this slice level could be used for all maneuvers without loosing the plane in dynamic.

**Fig. 5:**
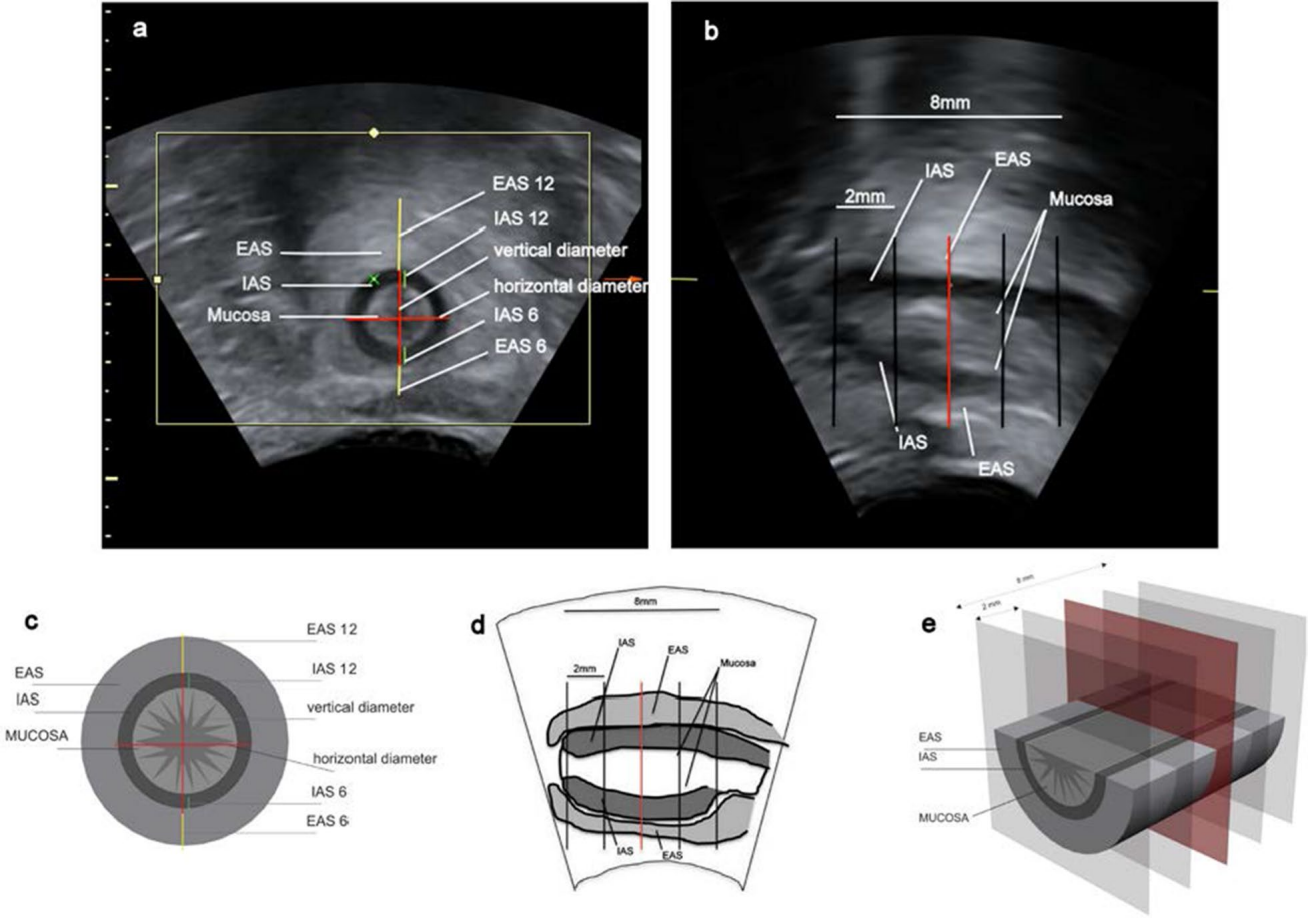
Measurements in PAUS volumes. **a** Presentation of the measurement positions for sphincter muscle thicknesses and diameters in coronal plane. **b** Definition of the plane for the measurements (red), 4 mm from the lowermost plane (left) where both sphincter muscles are seen completely the first time. **c** Schematic representation of measurement positions, **d** + **e** schematic representation of the defined plane for measurements

Different parameters were measured in this plane at rest (r) and during sphincter muscle contraction (c). Thickness of EAS and IAS were measured at 6 and 12 o’clock position. The vertical diameter was measured including the IAS at 6 o’clock and the IAS at 12 o’clock. Analogue measurement was done from 9 to 3 o’clock for the horizontal diameter (Fig. 5).

The means of the examiners measured values for vertical diameter at rest and contraction for PAUS were calculated to compare the different examinations. The mean value for vertical diameter during contraction was subtracted from the mean value of vertical diameter at rest for each patient, showing the distance the vertical diameter changed with contraction. This was compared with the mean values of the examiners DRESS contraction scores.

Study patients completed a questionnaire after their examinations to determine their subjective impressions of the clinical (DRE) and diagnostic examination (PAUS). Additionally study patients were asked about their stool behavior via questions modified from the Jorge–Wexner-Score. The Jorge–Wexner-Score is a tool to grade the severity of fecal incontinence. It contains questions about the frequency of loosing solid or fluid stools, flatulences, of using a pad and the influence on quality of life. The higher the frequency and thus the score is, the more severe is the fecal incontinence [2].

SPSS Version 24 was used for statistic analysis. Comparison of mean values was done with Wilcoxon-signed-rank test for paired data. As usual significance level has been *p* = 0.05 in all analysis. Pearson´s correlation was used to evaluate interrelation of the examinations.

## Results

In total 50 women underwent the examinations PAUS and DRE by two independent examiners. Thirty-five patients (70%) were included in the measurement part of the study. Fifteen (30%) were excluded due to following exclusion criteria: missing ultrasound pictures (n = 2), inadequate ultrasound picture quality plus sphincter defect (n = 1) and detected sphincter defects (n = 12). The mean age was 57 years with a range between 28 and 84 years (Table 1).

**Table 1:** Basic data of included women

	n	%	N
Age, mean, y	57		35
Number of given births, average	1.78		35
0 given births	4	11.43	35
1 vaginal delivery	12	34.29	35
2 vaginal deliveries	9	25.71	35
3 vaginal deliveries	5	14.70	35
4 vaginal deliveries	1	2.86	35
5 vaginal deliveries	1	2.86	35
1 Ceasarean section	2	5.71	35
2 Ceasarean section	1	2.86	35
No delivery information	3	8.57	35
Reported perineal tear			
I°	1	2.86	35
II°	0	0.00	35
III°	5	14.70	35
IV°	0	0.00	35
Urinary incontinence	23	65.71	35
Fecal incontinence	4	11.43	35

Twenty-four of the 50 questionnaires were excluded due to missing documents or incomplete answers given. Twenty-six questionnaires were evaluated for the patients’ subjective impressions about PAUS and DRE, but only 18 questionnaires belonging to the 35 included patients for PAUS and DRE were evaluated for stool behavior (Jorge–Wexner-Score).

Nine women had a perineal tear in their history. Thirteen sphincter defects were detected in the PAUS images and 12 of the women with detected sphincter defect had at least one vaginal birth. Concordance between history of a perineal tear and an ultrasound image detecting a sphincter defect was found in 3 women. Of the 9 women with reported FI, 5 had no structural abnormalities detected on ultrasound while sphincter defects were found in 4 patients.

Mean values for sphincter muscle thickness at rest and contraction measured in PAUS are presented in Table 2.

**Table 2:** Measurements of sphincter thickness at 6 and 12 o’clock and vertical and horizontal diameter in PAUS [in cm]

	Mean	Variance	SD	95% CI	n
a.					
rEAS 12	0.45	0.02	0.15	0.42–0.49	70
rEAS 6	0.41	0.03	0.17	0.37–0.45	70
rIAS 12	0.24	0.01	0.09	0.22–0.26	70
rIAS 6	0.18	0.00	0.06	0.17–0.20	70
r vert. diameter	1.67	0.06	0.24	1.62–1.73	70
r horiz. diameter	1.70	0.03	0.17	1.66–1.74	70
b.					
cEAS 12	0.44	0.02	0.14	0.40–0.47	70
cEAS 6	0.42	0.05	0.22	0.37–0.47	70
cIAS 12	0.24	0.01	0.09	0.22–0.26	70
cIAS 6	0.18	0.00	0.06	0.17–0.20	70
c vert. diameter	1.57	0.07	0.26	1.51–1.63	70
c horiz. diameter	1.68	0.05	0.22	1.63–1.73	70

Values of both examiners. a: PAUS measurements at rest, b: PAUS measurements during contraction. rEAS 12: external anal sphincter muscle at 12 o’clock position at rest, rEAS 6: external anal sphincter at 6 o’clock position at rest, rIAS 12: internal anal sphincter at 12 o’clock position at rest, rIAS 6: internal anal sphincter at 6 o’clock position at rest, r hor. diameter: horizontal diameter at rest (measured from IAS 9 o’clock to IAS 3 o’clock), r vert. diameter: vertical diameter at rest (measured from IAS 6 o’clock to IAS 12 o’clock). r = rest, c = contraction, legend for contraction is similar to the legend for rest

Table 3 shows the comparison of the sphincter muscle thickness at the different measurement positions and demonstrates significant differences between EAS 6 and 12 o’clock at rest, IAS 6 and 12 o’clock at rest, IAS 6 and 12 o’clock during contraction and the contraction diameters (horizontal–vertical). In these parameters sphincter thickness is larger at the 12 o’clock position than at 6 o’clock position and the horizontal diameter is greater than vertical diameter.

**Table 3:** Comparison of sphincter thickness at 6 and 12 o’clock and comparison of vertical and horizontal diameter in PAUS

Wilcoxon		Asympt. Sign.	6 < 12 or hor. < vert (%)	6 > 12 or hor. > vert (%)	6 = 12 or hor. = vert (%)
6–12 o’clock	rEAS	0.00	62.86	32.86	4.29
	rIAS	0.00	68.57	22.86	8.57
Horizontal–vertical	r diameter	0.20	42.86	57.14	0.00
6–12 o’clock	cEAS	0.06	52.86	40.00	7.14
	cIAS	0.00	68.57	27.14	4.29
Horizontal–vertical	c diameter	0.00	27.14	70.00	2.86

Wilcoxon-test. Separated evaluation of rest and contraction. EAS and IAS measurements compared between 6 and 12 o’clock and diameter compared between horizontal and vertical measurement. 6 < 12: percentage of a smaller value at 6 o’clock than value at 12 o’clock in percentage. 6 > 12: percentage of a greater value at 6 o’clock than value at 12 o’clock in percentage. 6 = 12: percentage of similar values at 6 o’clock and at 12 o’clock. Similar legend for diameter, hor.: horizontal, vert.: vertical, r: at rest, c: during contraction

The comparison of the examiners PAUS volumes by sphincter measurements showed only one significant difference, the vertical diameter at rest with means of 1.70 cm (examiner 1) and 1.64 cm (examiner 2). All other measurements in the ultrasound images at rest as well as during contraction showed no differences between examiners (Table 4).

**Table 4:** Comparison of the two examiners in PAUS

Wilcoxon	Asympt. Sign.		Asympt. Sign.
a. Rest		b. Contraction	
Examiner 1– examiner 2		Examiner 1– examiner 2	
rEAS 12	0.86	cEAS 12	0.64
rEAS 6	0.38	cEAS 6	0.73
rIAS 12	0.26	cIAS 12	0.90
rIAS 6	0.47	cIAS 6	0.80
r hor. diameter	0.49	c hor. diameter	0.46
r vert. diameter	0.04	c vert. diameter	0.31

Wilcoxon-test. a: PAUS measurements at rest, b: PAUS measurements during contraction

**Fig. 6:**
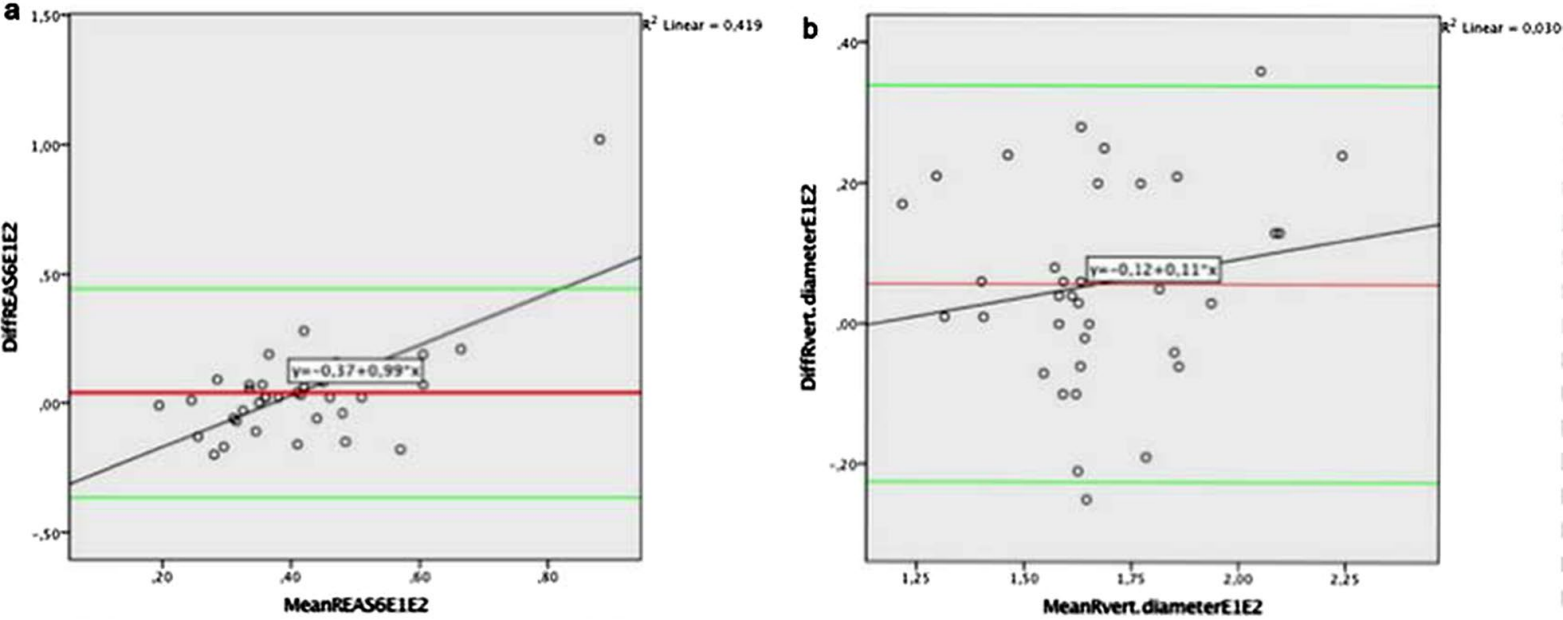
Examples of Bland–Altman-Plots to visualize agreement and differences between examiners for PAUS parameters at rest. **a** rEAS6 values with no difference between examiners, **b** r vertical diameter with significant interexaminers difference. DiffREAS6E1E2 = Differences in the measurement of EAS at rest at 6 o’clock between examiner 1 and examiner 2, MeanREAS6E1E2 = Mean values of the measurements of EAS at rest at 6 o’clock of examiner 1 and examiner 2. DiffRvert.diameterE1E2 = Differences in the measurement of vertical diameter at rest between examiner 1 and examiner 2, MeanRvert.diameterE1E2 = Mean values of the measurements of vertical diameter at rest of examiner 1 and examiner 2

Similar concordance and difference between the examiners is demonstrated with Bland–Altman-Plots (Fig. 6).

Although there is a significant difference between the examiners measurement for vertical diameter at rest, there is a highly significant positive correlation between these measurements with a Pearsons’ correlation coefficient of 0.82 with *p* = 0.01 (2-sided).

**Fig. 7:**
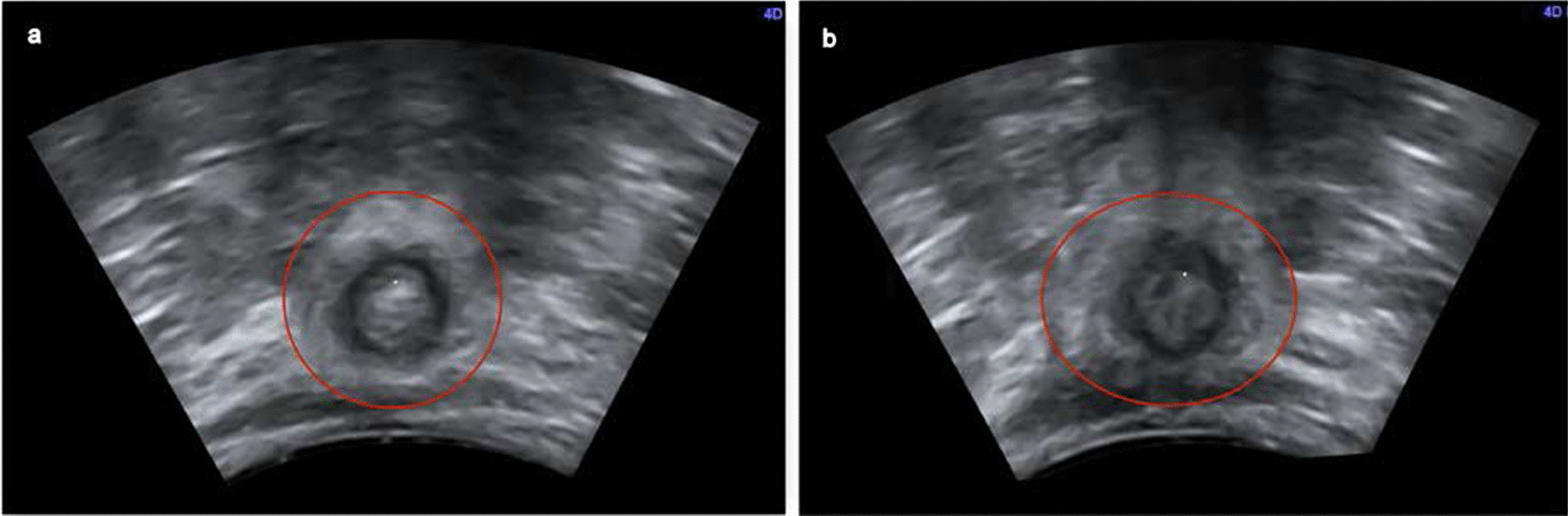
PAUS—comparison of rest and contraction. **a** At rest, **b** during contraction. Visible change of the anal canal shape (red) from round at rest (**a**) to oval during contraction (**b**)

The vertical diameter showed differences between rest (mean 1.67 cm) and contraction (mean 1.57 cm). In 79% of the patients, the contraction value of the vertical diameter was smaller than the value at rest. The horizontal diameter remained the same at rest and contraction. During contraction, the change in vertical diameter length without contraction of the horizontal diameter leads to the transformation of the anal complex from round to oval (Fig. 7).

All other measured data had no significant difference between rest and sphincter contraction as presented in Table 5.

**Table 5:** Comparison between rest and contraction of the sphincter thickness at 6 and 12 o’clock positions and the diameters in PAUS

Wilcoxon		Asympt. Sign.	c < r (%)	c > r (%)	c = r (%)
Rest—contraction	EAS 12	0.20	50.00	44.29	5.71
	EAS 6	0.77	51.43	41.43	7.14
	IAS 12	0.66	42.86	50.00	7.14
	IAS 6	0.90	47.14	42.86	10.00
	hor. diameter	0.12	54.29	35.71	10.00
	vert. diameter	0.00	78.57	20.00	1.43

Wilcoxon-test, r = rest, c = contraction. c < r: incidence of a smaller value during contraction than at rest in percentage. c > r: incidence of a greater value during contraction than value at rest in percentage. c = r: incidence of similar values during contraction and at rest in percentage

Table 6 compares the DRESS scores between the two examiners.

**Table 6:** DRESS values at rest and contraction separated by the examiners

	Mean	Median	SD	Variance	Minimum	Maximum	N
Examiner1							
rDRESS	2.54	3.00	0.77	0.59	0.00	5.00	35
cDRESS	2.40	3.00	0.95	0.89	0.00	5.00	35
Examiner2							
rDRESS	2.43	3.00	0.85	0.72	0.00	5.00	35
cDRESS	2.29	3.00	0.86	0.74	0.00	5.00	35

rDRESS: DRESS at rest, cDRESS: DRESS during contraction

Analysis showed no inter-examiner´s difference for the DRESS scores at rest as well as during contraction (Table 7a). Furthermore analysis showed no significant difference between rest and contraction scores (Table 7b).

**Table 7:** Comparison of the examiners DRESS scores (a), comparison between rest and contraction of examiners DRESS scores (b)

Wilcoxon		Asympt. Sign.
a. Examiner 2–examiner 1	r	0.17
	c	0.21
	r + c	0.06
b. Contraction (c)–rest (r)	E1	0.21
	E2	0.25
	E1 + E2	0.09

Wilcoxon-test; Examiner 1 (E1), examiner 2 (E2), examiner 1 and examiner 2 (E1 + E2), rest (r), contraction (c)

To show differences and similarities between PAUS and DRE we used following parameters for comparison, for PAUS the difference between rest and contraction of the vertical diameter (mean 0.11 cm, n = 70) and for DRE the contraction DRESS score (mean 2.34, n = 70). Results showed a slightly negative but not significant correlation between the distance the vertical diameter changed from rest to contraction and the contraction DRESS score with a Pearsons´ correlation coefficient of − 0.12 with *p* = 0.49 (2-sided).

There was one significant finding in the 26 complete questionnaires comparing DRE and PAUS. Women scored DRE as more embarrassing than PAUS. There was no significant difference between the examinations in being uncomfortable, unpleasant and painful and women’s expectation compared to reality.

As Table 8 shows, women found PAUS barely uncomfortable (mean 2.5), barely unpleasant (mean 2.38), hardly ever painful (mean 1.69) and barely embarrassing (mean 2.35). The subjects rated their experience with PAUS as at their expectation or better than their expectation (mean 0.58). All of the women in this study would allow a doctor to repeat this examination in the future. Women rated DRE as barely uncomfortable (mean 2.69), a little bit more unpleasant (mean 3.15), barely painful (2.0), a bit more embarrassing (mean 3.27) and their expectation about the examination matched with reality (mean 0.77). Twenty-two patients would allow their doctor to repeat DRE in the future without any limitations, 4 patients would agree to another DRE only exceptionally and no patient would deny it.

**Table 8:** Values of the questionnaire answers

	Mean	SD	Variance	Min	Max	n
a.						
1. PAUS uncomfortable?	2.50	2.20	4.82	1.00	10.00	26
2. PAUS unpleasant?	2.38	1.60	2.57	1.00	10.00	26
3. PAUS painful?	1.69	1.52	2.30	1.00	10.00	26
4. PAUS embarrassing?	2.35	1.90	3.60	1.00	10.00	26
5. PAUS expectation and reality?	0.58	0.50	0.25	0.00	2.00	26
6. Allowing repetition of PAUS in the future?	0.04	0.20	0.04	0.00	2.00	26
b.						
1. DRE uncomfortable?	2.69	1.74	3.02	1.00	10.00	26
2. DRE unpleasant?	3.15	2.29	5.26	1.00	10.00	26
3. DRE painful?	2.00	1.63	2.64	1.00	10.00	26
4. DRE embarrassing?	3.27	2.44	5.97	1.00	10.00	26
5. DRE expectation and reality?	0.77	0.59	0.35	0.00	2.00	26
6. Allowing repetition of DRE in the future?	0.15	0.37	0.14	0.00	2.00	26

a: answers of questions about PAUS, b: answers of questions about DRE. a + b: questions 1–4: scale for assessment was from 1 to 10, with 1 meaning not at all and 10 meaning extremely; question 5: scale was 0 = better, 1 = same and 2 = worse; question 6: possible answers were 0 = everytime, 1 = exceptionally and 2 = never. SD = standard deviation, min. = minimum of the scale, max. = maximum of the scale

In the questionnaire part according to the modified Jorge–Wexner-Score 11 of 18 women said that they have flatulence, which they cannot restrain. 6 women reported about the feeling to have urge to defecate that they cannot push back. A feeling of not being able to empty the bowel completely is felt by 9 patients. Suffering from loosing thin stools is the problem of 3 patients and 1 out of 18 patients is loosing solid stools sometimes.

## Discussion

In thirty-five women included in this study, perianal ultrasound (PAUS) was a reliable modality to diagnose anorectal function and disorders. The reproducibility of PAUS volumes was good, two independent examiners were able to perform PAUS examinations with similar 3D/4D-volumes. A difference was found in the sphincter thicknesses of EAS and IAS in PAUS between 6 and 12 o’clock positions with larger thickness at 12 o’clock position.. Images of the anal canal showed no difference between rest and contraction sphincter thicknesses of EAS and IAS, demonstrating that the muscle contractions are iso-volumetric. The vertical diameter decreased during contraction leading to an oval shape (Fig. 7).

Our results showed for the subjective examination DRE that it is also reproducible by different examiners.

For the comparison of PAUS and DRE we used the DRESS contraction score. It needs to be discussed that we did not include the DRESS resting scores, but we found that the DRESS resting score was independent of and did not correlate with the contraction scores of our patients. Comparison of PAUS and DRE showed a negative but not significant correlation between the distance the vertical diameter changed under contraction in PAUS and the DRESS contraction score. Interesting, we found that greater changes of the vertical diameter values during contraction measured in PAUS volumes did not correlate with a stronger contraction tone in DRE. In fact, shorter changes in the vertical contraction were associated with a stronger contraction tone in DRE. This assumes that the difference of the vertical diameter in PAUS is not a reliable parameter to grade rectal tone and sphincter contraction.

Contraction can only be visualized by the change in vertical diameter and by the disappearing mucosal rosette in ultrasound volumes. This supports our assumption that the pelvic floor muscles have a significant impact on the voluntary contraction, which is pulling the anal canal in cranial direction.

The difference in sphincter thicknesses of EAS and IAS in PAUS between 6 and 12 o’clock positions is interesting as our assumption was that sphincter muscles have a constant thickness in all positions. Our probe was placed on the anal opening and tilt 10°–20° in ventral direction, which rotates the axis of the plane. Because the anal canal is not a rigid tube and the probe tilted we assume that it is visualized in an oblique plane. Horizontal diameter was greater than the vertical diameter at rest and during contraction, but only contraction values showed significant differences. Probe rotation and the puborectal muscle with its activity in the voluntary contraction influence the vertical diameter.

Our measured EAS and IAS thicknesses at 12o’clock position have been in agreement with findings of other studies. We found comparable results to the reported values by Rao [1]. Good agreement have been found between our results and the reported by Beets-Tan et al., although they measured at 4o’clock position and used EAUS [18]. West et al. used 3D-EAUS and found EAS thicknesses at 6 and 12o’clock positions exactly the opposite to ours [19]. Differences may be attributed to the defined measurement positions and the different types of examinations. In regards to actual measurements, Williams et al. who used EAUS and eaMRI, measured values, which were at least doubled compared to ours. Differences to our study may be due to measuring positions in a horizontal line at midcanal level, younger patients and endoanal probes [20]. We doubt that we can reliably compare EAUS and PAUS, because the EAUS probe straightens the sphincter muscles and this changes structures. Additionally EAUS probes exist in different sizes, what could also explain the inhomogeneous results.

The shape changing to oval due to shorter vertical diameter under contraction although there is no difference in muscle thickness of IAS leads to the suggestion that there is an external influence of the surrounding area (pelvic floor with especially the puborectal muscle), which causes the change. The anal canal forms with the axis of the rectum an angle of approximately 90 degrees at rest. This angle becomes more acute during voluntary squeeze and more obtuse during defecation [1]. Pelvic floor muscles like EAS and puborectalis muscle belong to the few striated muscles of the body, which have spontaneous activity at rest and are never completely relaxed [21]. At rest the anus is closed by tonic activity of the IAS, a smooth muscle. During contraction maneuver patients can´t separate between muscles and do a full anal and pelvic floor muscle contraction, leading EAS and puborectalis muscle to reinforce this barrier. The sling of the puborectal muscle around the rectum creates a forward pull in the axis of the vertical diameter during contraction, resulting in an increasing anorectal angle and change of shape [1, 22]. Other studies as e.g. Olsen et al. found no difference between rest and contraction in EAUS measurements [23]. This could be explained by the fact that the EAUS probe has a round and rigid structure and can´t be modified by the changing surrounding muscle configuration. In reverse this could be leading to adaption of the muscles shape to the rigid structure of the endoanal ultrasound probe.

Compared to ultrasound, DRE is a subjective examination and needs some experience to provide an accurate score of the sphincter tone but is reproducible from different examiners as our results show. Studies before found out that DRE is a good and reliable examination and has a moderate to highly positive correlation with the objective measured sphincter tone by anorectal manometry [15, 24].

Even though PAUS demonstrates that the anal canal changes from round to oval during contraction, examiners doing DRE can only detect a circular contraction around their finger. Taverner et al. showed that different activities like phonation, elevated intra-abdominal pressure and voluntary contraction of pelvic floor muscles increase the EAS and puborectalis muscle activity [25]. DRE increases the sphincter tone reflective by touching perianal skin, due to highly sensible modulation [21, 25].

Taverner et al. and Weidner et al. found out with electromyography that puborectalis and EAS are tonically contracted. DRU increased sphincter tone during skin contact and dilatation of the canal but it diminished despite the presence of the finger. Voluntary contraction led to higher firing rates of motor units in EAS and puborectalis muscle, though the firing rate was higher in puborectalis muscle. Their findings showed that the levator ani muscle has larger more readily recruited motor units than the EAS [25, 26].

A huge difference between the methods is that DRE is a subjective impression and PAUS measurements are more objective. In the comparison of the methods we used a scoring-system of subjective impressions and objective measurable distance changes. The DRESS-Score is the try to give a subjective impression a more objective and comparable value. But also measurements in ultrasound volumes have a small subjective component as examination and measurement are always dependent on the examiner. Differences of the examinations are that DRE gives an impression of the sphincter muscle function by muscle tone and PAUS presents the structures of the sphincter muscle and surrounding area. Similar maneuvers were performed in both methods.

DRE is clinically used to get an impression of muscle tone and palpate for irregularities. Disadvantage of DRE is that the subjective impression of muscle tone is only documentable with scores what makes exact comparison in follow-up visits harder and giving bio-feedback for patients is not possible.

Potential clinical value of PAUS is to be a standardized screening tool postpartum to detect asymptomatic or symptomatic sphincter defects or when FI symptoms occur. Early detection of lesions may improve patient outcomes [8, 27]. PAUS could a be fundamental diagnostic tool to fill memory gaps of perineal tear history or to proof residuals with present symptoms if there is no history of perineal tear known. In addition, PAUS could be helpful to determine the potential factors leading to FI such as structural sphincter defects, prolapse, hemorrhoids or fecal impaction [8, 27]. PAUS can be used for documentation of volumes, which leads to better comparability in follow up visits. Another potential clinical value of PAUS could be the possibility to give patients a bio-feedback about structure and function of the anal sphincter muscles. It could also be a helpful support in the consultation of birth planning after a previously occurred perineal tear grade III or IV.

PAUS and DRE are perfect supplementary examinations, which improve the examiners four-dimensional impression. PAUS is, like PUS, widely available, less expensive and obviously better tolerated by patients than DRE or EAUS [8, 11, 12].

The mean age (57 years) of our study was not representative for the whole population, but reflects the age of patients suffering from FI [3, 4]. There is also a wide age range in study patients. For better comparison of the patients and the measurement results further studies would improve by a more homogenous group to rule out influence of variables as age or birth modality. Literature contains different statements about the influence of age on sphincter thickness. Starck et al. found that there is no correlation between age and sphincter measurements in EAUS, Murad-Regadas et al. found a thicker IAS in older nulliparous women compared to younger nulliparous women, but in women with vaginal or cesarean birth they found no influence of age on sphincter thickness [28, 29]. Frudinger et al. showed a significant correlation in EAUS between age and sphincter thickness, positive correlation for IAS, negative for EAS in nulliparous women [30]. Further studies should investigate and evaluate this question with PAUS as current data is mostly available with EAUS data.

Our study is limited by the fact that we only included patients without sphincter defects in ultrasound and had a small sample size. There was no preevaluation of the patient group in our study. In future a more homogenous patient group should be defined before starting with measurements to minimize the number of excluded patients. Also improvement of ultrasound technology will help to improve the quality of samples. The questionnaire maybe was too long and this minimizes the motivation of patients to finish it. To improve this, shorter questionnaires could help. Generally it needs more reconnaissance and awareness about the relevance and importance of answering medical questionnaires for research in medicine.

The clinical power of small sample sizes is low. Studies with larger sample sizes are necessary to confirm our findings. We see our study as a work to get a basis for further studies. Clinically it is a clue to work with and to investigate in future studies in this topic, as there is still a huge part in the physiology and complexity of the pelvic floor we do not understand completely.

Another limitation is that only one examiner performed the measurements in the PAUS images.

## Conclusion

PAUS is reproducible and an accurate and appropriate imaging tool to visualize the anal canal. PAUS is easy to learn, low cost and widely available. Patient acceptance was positive. Resting tone of the sphincter muscles is important for continence and sphincter thickness does not change through maneuvers. PAUS images demonstrate that sphincter contraction is iso-volumetric, the disappearing rosette under contraction and changing anal canal shape to oval are the only visible activities. PAUS visualizes relevant perianal structures and can diagnose abnormalities suspected on DRE. However PAUS cannot accurately measure rectal tone. DRE remains the gold standard to determine patients’ rectal tone and PAUS is the ideal additional tool to the DRE. Apparently continence is an interaction between pelvic floor and sphincter muscle tone and further investigations should proof which effect a sphincter defect has on this interaction.

## Data Availability

The datasets used and analyzed during the current study are available from the corresponding author on reasonable request.

## Abbreviations

PAUS: Perianal ultrasound
DRE: Digital-rectal examination
FI: Fecal incontinence
PUS: Perineal ultrasound
EAUS: Endoanal ultrasound
eaMRI: Endoanal magnetic resonance imaging
TVT slings: Tension-free vaginal tape
DEGUM: Deutsche Gesellschaft für Ultraschall in der Medizin e.V.
AGUB: Arbeitsgemeinschaft für Urogynäkologie und plastische Beckenbodenrekonstruktion e.V.
DRESS: Digital rectal exam scoring system
TUI: Tomographic ultrasound imaging
EAS: External anal sphincter muscle
IAS: Internal anal sphincter muscle
CI: Confidence interval
SD: Standard deviation
vert. diam.: Vertical diameter
horiz. diam.: Horizontal diameter
c: Contraction
r: Rest
asympt. Sign.: Asymptotic significance
